# Retroconversion is a minor contributor to increases in eicosapentaenoic acid following docosahexaenoic acid feeding as determined by compound specific isotope analysis in rat liver

**DOI:** 10.1186/s12986-017-0230-2

**Published:** 2017-11-28

**Authors:** Adam H. Metherel, Raphaël Chouinard-Watkins, Marc-Olivier Trépanier, R. J. Scott Lacombe, Richard P. Bazinet

**Affiliations:** Department of Nutritional Sciences, Faculty of Medicine, University of Toronto, 150 College St., Room 307, Fitzgerald Building, Toronto, ON M5S 3E2 Canada

**Keywords:** Retroconversion, Liver, Metabolism, Eicosapentaenoic acid, Docosahexaenoic acid, Alpha-linolenic acid

## Abstract

Dietary docosahexaenoic acid (DHA, 22:6n-3) not only increases blood and tissue levels of DHA, but also eicosapentaenoic acid (EPA, 20:5n-3). It is generally believed that this increase is due to DHA retroconversion to EPA, however, a slower conversion of α-linolenic acid (ALA, 18:3n-3) derived EPA to downstream metabolic products (i.e. slower turnover of EPA) is equally plausible. In this study, 21-day old Long Evans rats were weaned onto an ALA only or DHA + ALA diet for 12 weeks. Afterwards, livers were collected and the natural abundance ^13^C-enrichment was determined by compound specific isotope analysis (CSIA) of liver EPA by isotope ratio mass-spectrometry and compared to dietary ALA and DHA ^13^C-enrichment. Isotopic signatures (per mil, ‰) for liver EPA were not different (*p* > 0.05) between the ALA only diet (−25.89 ± 0.39 ‰, mean ± SEM) and the DHA + ALA diet (−26.26 ± 0.40 ‰), suggesting the relative contribution from dietary ALA and DHA to liver EPA did not change. However, with DHA feeding estimates of absolute EPA contribution from ALA increased 4.4-fold (147 ± 22 to 788 ± 153 nmol/g) compared to 3.2-fold from DHA (91 ± 14 to 382 ± 13 nmol/g), respectively. In conclusion, CSIA of liver EPA in rats following 12-weeks of dietary DHA suggests that retroconversion of DHA to EPA is a relatively small contributor to increases in EPA, and that this increase in EPA is largely coming from elongation/desaturation of ALA.

## Introduction

Dietary docosahexaenoic acid (DHA, 22:6n-3) is primarily obtained from marine and seafood sources [1, 2], and increased consumption of n-3 polyunsaturated fatty acids (n-3 PUFA) are known to increase blood and tissue n-3 PUFA levels. These changes in tissue n-3 fatty acids can regulate numerous biological processes including many that are related to brain function [3]. Dietary DHA not only increases blood and tissue DHA concentrations, but has repeatedly been shown to increase eicosapentaenoic acid (EPA, 20:5n-3) – a metabolic precursor in the biosynthesis of DHA – as well, with very recent studies in humans [4], rats [5], mice [6] and pigs [7] demonstrating this response. In vitro evidence for retroconversion appears to be mediated by a round of peroxisomal β-oxidation in combination with saturation by Δ3,Δ2-enoyl-CoA isomerase, Δ3,5,Δ2,4-dienoyl-CoA isomerase and 2,4-dienoyl-CoA reductase [8–13]. Recently, Park et al. [9] demonstrated retroconversion in breast epithelial (MCF7), liver (HepG2), neuronal (SK-N-SH) and retinal (Y79) cancer cell lines, and their results suggest that although retroconversion is present in each tissue, retroconversion is higher in the non-neuronal tissue.

Although the evidence for retroconversion of DHA to EPA in vitro is clear, much of the earlier evidence for retroconversion of DHA to EPA in vivo was supported through dietary feeding studies where dietary DHA also increases blood or tissue EPA levels [14], or from oral doses of ^13^C-DHA tracers [15]. In dietary DHA feeding studies an assumption is often made that all increases in EPA are a result of retroconversion [14, 16], and these studies estimate that 7–14% of DHA is retroconverted to EPA; however, the source of EPA in such feeding studies cannot be verified to be DHA or a precursor to EPA such as α-linolenic acid (ALA, 18:3n-3). In the latter case, the desaturation and elongation of ALA may be slowed at EPA such that the turnover of EPA via elongation/desaturation, eicosanoid production or even β-oxidation may be downregulated. Oral doses of ^13^C-DHA indicate a much lower rate of retroconversion based on plasma appearance of ^13^C-EPA, ^13^C-docosapentaenoic acid (DPAn-3, 22:5n-3) and ^13^-ALA to be between 0.7 and 4.3% [15, 17, 18] in humans and 9% in rats [15]. Although isotopic evidence for retroconversion of DHA to EPA discussed above appears strong, it remains unclear what proportion of the increases in EPA following DHA intake in vivo, if any, may be attributed to either increased retroconversion or a decreased EPA turnover. Recently, our lab has exploited differences in natural ^13^C/^12^C abundance between dietary ALA and DHA sources combined with compound specific isotope analysis (CSIA) by isotope-ratio mass-spectrometry (IRMS) to determine the origin of mouse brain DHA [19].

Materials isolated from C_3_ plants such as ALA, are isotopically depleted (−23‰ to −32‰) in ^13^C compared to materials isolated from C_4_ plants (−10‰ to −16‰) [20]. Furthermore, the fractionation of carbon isotopes from aquatic organism results in ^13^C enrichment of aquatic materials such as DHA that differs slightly (−16‰ to −22%) from both C_3_ and C_4_ plants [19, 21]. These natural differences in isotopic enrichment of ALA and DHA allows for determination of the dietary source of individual fatty acids in the PUFA biosynthesis pathway. Therefore, through the determination of the natural ^13^C/^12^C liver EPA abundance levels, the purpose of the current study is to CSIA to identify the source of liver EPA following 12 weeks of an ALA only diet compared to a DHA + ALA diet. Briefly, we identified no differences in the natural ^13^C/^12^C abundance in liver EPA between ALA only and DHA + ALA diets despite nearly 5-fold higher total liver EPA concentration on the DHA + ALA diet. We further determined that ALA was the primary source for the increase in liver EPA following DHA feeding; suggesting that slower metabolism of EPA – and not retroconversion from DHA – may be the primary mechanism responsible for the increased EPA. The present analysis was performed as sub-analysis from a larger DHA supplementation study that has yet to be published.

## Methods

### Animals

All experimental procedures were performed in agreement with the policies set out by the Canadian Council on Animal Care and were approved by the Animal Ethics Committee at the University of Toronto (Protocol # – 20011797). The animals and data used for this study were taken as a sub-analysis of a larger animal DHA supplementation protocol. Long Evans dams with male non-littermate pups were ordered from Charles River Laboratories (St. Constant, QC, Canada). Upon arriving at the University of Toronto, the dams and pups were acclimated for 3 days and at 21-days old the pups were randomly assigned and weaned onto either a 2% ALA diet (*n* = 6) or a 2% ALA +2% DHA diet (*n* = 6) for 12 weeks. At 15-weeks old, animals were euthanized by carbon dioxide asphyxiation and liver samples were collected from the right medial lobe, flash-frozen in liquid nitrogen and stored at −80 °C until analysis.

### Diets

The diets were modified from the AIN-93G custom low n-3 rodent diets (Dyets, Inc., Bethlehem, PA, USA). The diets contained 10% lipids by weight with the fat content being 32.8% safflower oil, 63.2% hydrogenated coconut oil and 4% added fatty acid ethyl ester oils. The added oils were 2% oleate ethyl ester (Nu-Chek Prep, Inc., Elysian, MN, USA) and 2% ALA ethyl ester (gift from BASF Pharma, Callanish Ltd., Isle of Lewis, UK) in the ALA diet, and in the DHA diet the added oleate ethyl ester was replaced by 2% DHA ethyl ester (gift from BASF Pharma, Callanish Ltd.). Oleate ethyl ester was included in the ALA diet to maintain the total fat content equivalent to that of the DHA diet. Each oil was determined to be >98% pure by gas chromatography-flame ionization detection (GC-FID).

### Lipid extraction

Total lipid extracts (TLE) were obtained from approximately 50 mg of liver tissue by a method modified from Folch, Lees and Sloane-Stanley [22]. Briefly, liver tissue was homogenized with a Polytron benchtop homogenizer (Brinkman Instruments, Toronto, ON, Canada) in 6 mL of 2:1 chloroform methanol containing 30 mg of docosatrianoic acid (22:3n-3) ethyl ester as internal standard, and vortexed. A 0.88% potassium chloride aqueous buffer (1.75 mL) was added, and samples were inverted twice and centrifuges at 500 g for 10 min to separate the lipid-containing chloroform phase from the aqueous phase. The chloroform lipid-containing layer was collected and stored in a new test tube.

### Transmethylation and GC-FID

An aliquot of the TLE was collected, dried under nitrogen and transmethylated via a method adapted from Morrison and Smith [23] using 1 mL 14% boron trifluoride in methanol and 0.3 mL hexane at 100 °C for 1 h. Hexane and water (1 mL each) were added, vortexed and centrifuged at 500 g for 5 min. The hexane layer containing fatty acid methyl esters (FAMEs) was collected, evaporated under nitrogen, reconstituted in hexane and stored in GC vials for analysis by GC-FID as previously described [19]. Peaks were identified by retention times through comparison to an external FAME standard (GLC-569, Nu-Chek Prep Inc.). After running on GC-FID, samples were re-capped and stored at −80°C for CSIA analysis by IRMS.

### Isotopic analysis

CSIA of isolated FAMEs was performed by GC-IRMS, as described previously in detail [19]. Briefly, FAMEs (2 μL) were injected in splitless mode using a TriPlus RSH autosampler (Thermo Scientific, Bremen, Germany) onto the SP-2560 capillary column described earlier interfaced in a Trace 13,010 GC (Thermo Scientific). Complete resolution of analyte peaks of interest from surrounding peaks is imperative for high-precision CSIA by GC-IRMS [24]. Therefore, co-elution of EPA with 24:0 using the oven temperature program described previously necessitated an alternate temperature program for complete resolution of EPA from surrounding peaks by GC-IRMS. Complete baseline separation of EPA was achieved with the following oven temperature program: initial temperature of 60 °C with an immediate ramp of 15 °C/min to 180 °C and an immediate ramp to 1.5 °C/min to 240 °C and an 18 min hold. Column flow rate was set to 1.2 mL/min.

### Isotopic normalization

Isotopic abundance data collected by IRMS was normalized and converted to the international carbon isotope reference scale, Vienna Peedee Belemnite (VPBD), by multi-point linear normalization and reported as per mil (‰) [25]. Certified calibrated 20-carbon FAME reference materials USGS70, USGS71 and USGS72 were injected at least once each during the sequence. Linear regression of measured values versus true values (−30.53 ± 0.04, −10.50 ± 0.03, and −1.54 ± 0.03 ‰ for USGS70, USGS71, and USGS72, respectively) was used to generate the normalizing equation to report δ^13^C values for all data. R^2^ values for all normalizing equations were >0.9998.

### Methylation correction

Isotopic analysis by GC-IRMS provides data on CO_2_ produced from the combustion of individual FAMEs, and therefore measurements include the isotopic contribution of carbon from the derivatized methyl group. To account for the contribution of the derivatized carbon, a methyl correction calculation was performed as previously described in detail [19].

### Estimation of dietary source of FAMEs

Isotopic signatures in the ALA only diet have previously been determined to −28.22 ± 0.29 ‰ for ALA and −30.44 ± 0.09 ‰ for linoleic acid (LNA, 18:2n-6) [19], and in the DHA + ALA diet we determined isotopic signatures at −22.30 ± 0.27 ‰ for DHA, −28.54 ± 0.22 ‰ for ALA and −30.77 ± 0.08 ‰ for LNA (Table 1). Since ALA and DHA are the only n-3 fatty acids present in the diets, we can compare these dietary signatures to those determined in the liver and estimate the proportion of a specific FAME, presently EPA, that is derived from either the ALA source or the DHA source [26]. The equation is as follows:

1 $$ {\updelta_{\mathrm{EPAliver}}}^{13}\mathrm{C}=\mathrm{x}\ast {\updelta_{\mathrm{ALAdiet}}}^{13}\mathrm{C}+\left(1\hbox{--} \mathrm{x}\right)\ast {\updelta_{\mathrm{DHAdiet}}}^{13}\mathrm{C} $$

**Table 1:** Dietary fatty acid composition and isotopic signatures

Fatty Acid	ALA	DHA + ALA
	weight % in total FAs	
C 10:0	4.68 ± 0.01	4.26 ± 0.03
C 12:0	30.8 ± 0.2	28.0 ± 0.2
C 14:0	12.0 ± 0.1	10.8 ± 0.1
C 16:0	8.54 ± 0.02	8.31 ± 0.03
C 18:0	8.05 ± 0.05	7.42 ± 0.03
SFAs	64.3 ± 0.2	59.2 ± 0.2
C 18:1n-7	0.26 ± 0.01	0.30 ± 0.01
C 18:1n-9	7.74 ± 0.04	6.66 ± 0.04
MUFAs	8.18 ± 0.05	7.15 ± 0.05
C 18:2n-6	24.7 ± 0.1	29.4 ± 0.1
*δ* ^*13*^ *C*_*18:2n-6*_	*−30.44 ± 0.09 ‰*	*−30.77 ± 0.08 ‰*
C 20:4n-6	n.d.	0.07 ± 0.0003
N-6	24.7 ± 0.1	29.5 ± 0.1
C 18:3n-3	2.07 ± 0.01	1.91 ± 0.01
*δ* ^*13*^ *C*_*18:3n-3*_	*−28.22* ± *0.17* ‰	*−28.54 ± 0.22 ‰*
C 20:5n-3	0.01 ± 0.001	0.01 ± 0.0003
C 22:6n-3	0.01 ± 0.001	1.72 ± 0.01
*δ* ^*13*^ *C*_*22:6n-3*_	*–*	*−22.30* ± *0.27* ‰
N-3	2.1 ± 0.01	3.64 ± 0.02

Values are mean ± SEM, *N* = 3

Where x = the proportion of liver EPA from dietary ALA, 1 – x = the proportion of liver EPA from dietary DHA. These proportions can then be applied to total liver EPA concentrations to estimate the absolute amounts of liver EPA derived from either ALA or DHA. The equations are as follows:

2 $$ {\left[\mathrm{EPA}\right]}_{\mathrm{Total}}={\left[\mathrm{EPA}\right]}_{\mathrm{ALAdiet}}+{\left[\mathrm{EPA}\right]}_{\mathrm{DHAdiet}} $$

where,

3 $$ {\left[\mathrm{EPA}\right]}_{\mathrm{ALAdiet}}=\mathrm{x}\ast {\left[\mathrm{EPA}\right]}_{\mathrm{Total}} $$

4 $$ {\left[\mathrm{EPA}\right]}_{\mathrm{DHAdiet}}=\left(1\hbox{--} \mathrm{x}\right)\ast {\left[\mathrm{EPA}\right]}_{\mathrm{Total}} $$

### Statistics

All statistical analyses were performed with IBM SPSS Statistics 24 software (IBM, Armonk, New York). Differences between diets for total fatty acid concentrations and proportion of EPA from ALA and arachidonic acid (ARA, 20:4n-6) from LNA were determined by Student’s t-test. Differences in concentration estimates of sources of liver EPA and ARA were assessed by two-way ANOVA followed by Student’s t-test between diets and paired t-test between sources. Significance for all statistical analyses was determined at *p* < 0.05. All data is presented as mean ± SEM.

## Results

### Liver fatty acid concentrations

**Fig. 1:**
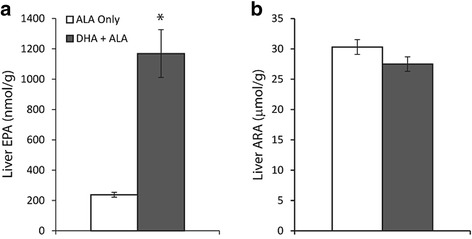
Liver concentrations of (**a**) eicosapentaenoic acid and (**b**) arachidonic acid following 12 weeks ingestion of 2% ALA or 2% DHA + 2% ALA diet in male Long Evans rats. * – represents statistically significant liver concentration between dietary groups as determined by significant Student’s t-test, *p* < 0.05. *N* = 6, mean ± SEM. ALA – α-linolenic acid, 18:3n-3; ARA – arachidonic acid, 20:4n-6; DHA – docosahexaenoic acid, 22:6n-3; EPA – eicosapentaenoic acid, 20:5n-3

Total liver fatty acid concentrations (Fig. 1) were determined for EPA and ARA and are presented in nmol/g and μmol/g, respectively. EPA increased (*p* < 0.05) from 238 ± 16 nmol/g on the ALA only diet to 1169 ± 158 nmol/g on the DHA + ALA diet, an increase of nearly 4-fold. In addition, liver DPAn-3 concentrations increased (*p* < 0.05) from 581 ± 41 nmol/g to 979 ± 99 nmol/g on the ALA only and DHA + ALA diet, respectively (data not shown). No difference (*p* = 0.13) in ARA concentration was determined between the ALA diet (30.1 ± 1.2 μmol/g) and DHA + ALA (26.8 ± 1.1 μmol/g).

### Eicosapentaenoic acid and arachidonic acid isotopic signatures

**Fig. 2:**
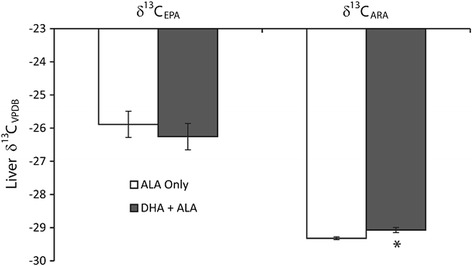
Liver carbon isotope signatures for eicosapentaenoic acid and arachidonic acid in male Long Evans rats following 12 weeks ingestion of 2% ALA or 2% DHA + 2% ALA diet in male Long Evans rats. * – represents significantly different liver carbon isotope signatures between dietary groups as determined by significant Student’s t-test, *p* < 0.05. *N* = 6, mean ± SEM. ALA – α-linolenic acid, 18:3n-3; ARA – arachidonic acid, 20:4n-6; DHA – docosahexaenoic acid, 22:6n-3; EPA – eicosapentaenoic acid, 20:5n-3; VPDB – Vienna Peedee Belemnite

After 12 weeks of dietary feeding, there was no change in the isotopic signature of liver EPA with values determined to be −25.89 ± 0.39 ‰ and −26.26 ± 0.40 ‰ for the ALA only and DHA + ALA diets, respectively (Fig. 2). Conversely, liver ARA on the ALA only diet was less enriched (*p* = 0.017) in ^13^C at −29.32 ± 0.04 ‰ compared to the DHA + ALA only diet at −29.07 ± 0.08 ‰ (Fig. 2).

### Contribution of dietary ALA to EPA and dietary LNA to ARA

**Fig. 3:**
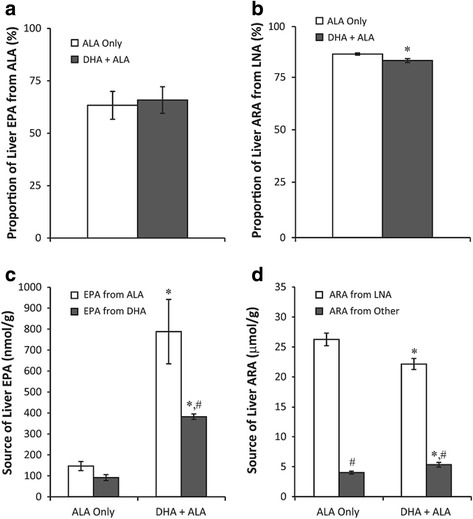
Estimates of (**a**) percent of liver EPA derived from ALA, (**b**) absolute concentration of liver EPA from ALA and DHA sources, (**c**) percent of liver ARA derived from LNA and (**d**) absolute concentration of liver ARA derived from LNA and other sources. * - represents significant differences between diets for proportion of liver EPA from ALA (**a**) and ARA from LNA (**b**) as determined by Student’s t-test. * - represents significant differences between diets by Student’s t-test and # represents significant differences between sources of EPA and ARA by paired t-test following significant interaction effect by two-way ANOVA (**c** and **d**), *p* < 0.05. *N* = 6, mean ± SEM. **c** Interaction = 0.036, Diet <0.0001, Source = 0.008; **d** Interaction = 0.001, Diet = 0.07, Source <0.0001. ALA – α-linolenic acid, 18:3n-3; ARA – arachidonic acid, 20:4n-6; DHA – docosahexaenoic acid, 22:6n-3; EPA, eicosapetaenoic acid, 20:5n-3; LNA – linoleic acid, 18:2n-6

Using Eq. 1 described earlier, we estimated that there is no difference (*p* > 0.05) in the proportion of liver EPA that was derived from dietary ALA sources in the ALA only diet (60.6 ± 6.6%) compared to the DHA + ALA diet (63.4 ± 6.4%) (Fig. 3a). Conversely, a higher (*p* < 0.05) proportion of liver ARA was estimated to be derived from dietary linoleic acid (LNA, 18:2n-6) in the ALA only diet (86.7 ± 0.5%) as compared to the DHA + ALA diet (80.6 ± 0.9%) (Fig. 3b). Utilizing these proportional estimates, the absolute contribution of ALA versus DHA sources to liver EPA concentrations, and the absolute contribution of LNA or other sources to liver ARA concentration were estimated using Eq. 2 (Fig. 3c and d, respectively). Liver concentration of EPA derived from dietary ALA sources increased (*p* < 0.01) from 147 ± 22 nmol/g on the ALA only diet to 788 ± 153 nmol/g on the DHA + ALA diet. Similarly, liver EPA derived from dietary DHA sources (or more ^13^C–enriched sources) was also higher (*p* < 0.001) on the DHA + ALA diet (382 ± 13 nmol/g) compared to the ALA only diet (91 ± 14 nmol/g). These contributions represent an apparent retroconversion rate of 4.5 ± 0.6% as a result of dietary DHA + ALA feeding. Liver concentration of ARA derived from LNA was higher (*p* < 0.05) in the ALA only diet (26.3 ± 1.1 μmol/g) compared to the DHA + ALA diet (22.1 ± 0.9 μmol/g). Conversely, a lower (*p* < 0.05) proportion of ARA was derived from non-LNA sources in the ALA only diet (4.0 ± 0.2 μmol/g) compared to the DHA + ALA diet (5.3 ± 0.4 μmol/g).

## Discussion

Presently, we performed a sub-analysis on a dataset obtained from a larger DHA supplementation study in Long Evans rats. Liver TLE were analyzed by GC-FID for concentration and by GC-IRMS for CSIA of EPA and ARA to determine the effects of 12 weeks of DHA feeding on the dietary contribution to liver EPA, and ultimately the contribution of apparent retroconversion of DHA to EPA. Briefly, we demonstrated that adding 2% dietary DHA to a 2% ALA diet does not change the isotopic signature of liver EPA. However, due to the nearly 4-fold increase in total liver EPA we estimated that the absolute amount of liver EPA derived from both dietary ALA and DHA are higher following DHA + ALA feeding compared to ALA feeding only. Although it is not clear what lipid fraction liver EPA is being incorporated into presently, previous studies have shown that following 14 weeks DHA supplementation in humans plasma EPA concentration increases in the phospholipid fraction with no changes in EPA concentration in plasma triacylglycerols or cholesteryl esters [27]. Overall, our carbon isotopic analysis suggests that while retroconversion of dietary DHA to liver EPA may be occurring, it is a relatively small contributor to increases in EPA compared to a reduced turnover of EPA.

**Fig. 4:**
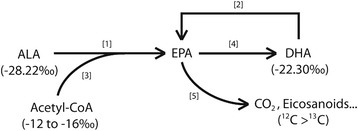
Representation of theoretical carbon flow influencing ^13^C enrichment of EPA in the ALA + DHA diet. [1] Elongation and desaturation of dietary ALA (−28.22‰), [2] retroconversion of dietary DHA (−22.30‰), [3] 2 carbons via acetyl-CoA provided from glycolysis (−12 to −16‰) for elongation of ALA, [4] slower elongation/desaturation of EPA to DHA, [5] preferential β-oxidation of lighter ^12^C isotopes from EPA and additional EPA metbaolism. ALA – α-linolenic acid, 18:3n-3; DHA – docosahexaenoic acid, 22:6n-3; EPA – eicosapentaenoic acid, 20:5n-3

For the purpose of identifying any potential underlying contribution to liver EPA from carbon recycling through glycolysis, we included an assessment of the carbon isotopic abundance of ARA which is a product of the shared PUFA synthesis pathway, but of the n-6 portion. This is important since both diets include the 18-carbon n-6 dietary equivalent to ALA, with 24–29% LNA (Table 1), but no dietary equivalent to DHA. Following 12 weeks ingestion of the DHA + ALA diet, liver ARA was significantly more enriched compared to the ALA only diet, suggesting that a more enriched source of ARA may be present, independent of retroconversion. Dietary carbohydrates present in our diets are isolated from corn, which is a C_4_ plant. C_4_ plants are highly enriched in ^13^C with carbon isotopic signatures of between −10 and −16 ‰ compared to C_3_ plants with signatures between −23 and −32 ‰ [20]. As such, acetyl-CoA produced from glycolysis can be used in the chain elongation of LNA to ARA, thereby increasing the ^13^C enrichment of ARA. Alternatively, β-oxidation favoring the isotopically lighter carbons [28] or carbon recycling from DHA into ARA through the acetate pool [29] could further explain the ^13^C-enrichment of ARA following the DHA + ALA diet. Finally, the presence of a very small amount (0.07 ± 0.0003%) of ARA in the DHA + ALA diet could influence the results; however, levels were too low to determine isotopic signatures. The potential ^13^C enrichment of liver ARA from acetyl-CoA, β-oxidation or acetate is important when discussing the apparent retroconversion of DHA to EPA as similar contributions may be present for EPA, and may therefore overestimate the determined EPA contribution from DHA. In fact, liver EPA enrichment on the ALA only diet appears to indicate that only 61% of liver EPA is coming from ALA suggesting a more enriched source of carbons for EPA on the ALA only diet, and indicates a significant contribution from the acetyl-CoA pool via glycolysis or preferential β-oxidation. A summary of the potential carbon contributions to EPA is provided in Fig. 4.

Despite these findings, no differences in the estimated proportion of liver EPA derived from ALA or DHA were determined between ALA only and DHA + ALA diets; however, the estimated absolute contribution to liver EPA from dietary ALA and DHA were increased by 4.4 and 3.2-fold following the DHA + ALA diet, respectively. This suggests that although there may be more EPA being produced from DHA, it does not appear to be a result of an upregulation of enzymes in the retroconversion pathway. In support, the expression of 2,4-dienoyl-CoA reductase 2, an enzyme involved in the removal of one double bond from DHA for retroconversion [9], shows no effect of DHA supplementation in pigs fed increasing dietary DHA [7]. However, they also showed no effect on elongation of very long-chain 5 (Elovl5), one of two enzymes (the other being Elovl2) responsible for the elongation of EPA to DPAn-3. Our IRMS data indicates that the increase in liver EPA concentration is not the result of an increase in proportional contribution from DHA retroconversion, suggesting that EPA elongation into DPAn-3 may in fact be lower suggesting a downregulation of Elovl5 and/or Elovl2. Importantly, β-oxidation and/or eicosanoid production from EPA among other pathways may also be downregulated. However, although liver DPAn-3 concentrations are also significantly higher on our DHA + ALA diet, the increase of only 69% is much less pronounced than the 490% higher EPA levels on the DHA + ALA diet, and is supportive of a reduced elongation of EPA to DPAn-3. Furthermore, dietary DHA supplementation on a high-fat diet significantly reduced Elovl5 expression in rats, where Elovl2 was lower but not significantly [30]. Low affinity of Elovl2 for 18-carbon PUFAs combined with high affinity for 20-carbon PUFAs – particularly n-3 s [31] – would implicate Elovl2 as a primary contributor to lower EPA turnover/elongation with DHA feeding. Interestingly, Elovl2 knockout mice have higher liver EPA levels compared to wild type mice [32] in a manner similar to that of dietary DHA shown in our study. Future studies should be aimed at assessing the role of Elovl2 and Elovl5 in increased EPA concentrations following DHA feeding.

The increase in estimated EPA via retroconversion shown presently represents 4.5 ± 0.6% of DHA being retroconverted to EPA after 12 weeks of feeding, and is lower than the 13.9 ± 2.9% calculated if all increases in EPA were assumed to be due to retroconversion. The latter calculation has been implemented previously with apparent retroconversion of DHA to EPA determined to be between 9 and 11% [14, 33, 34] in human plasma/serum. Our values are comparable to apparent retroconversion determinations of between 0.7 and 4.3% in human plasma 28 days following an oral dose of isotopically labeled ^13^C–DHA from individuals not supplemented with DHA [17, 18], and 5 months of fish oil supplementation does not increase the apparent retroconversion to EPA as determined by the appearance of plasma ^13^C-EPA [35]. Furthermore, if retroconversion was solely responsible for the higher liver EPA with DHA feeding, liver EPA would become isotopically enriched and more closely resemble the enrichment of dietary DHA (22.30 ± 0.27‰), and at 25.89 ± 0.39‰ and −26.26 ± 0.40‰ for the two diets, this is not the case.

## Conclusions

By utilizing CSIA of liver EPA by IRMS following 12-weeks supplementation of an ALA only diet or a DHA + ALA diet we have determined that the primary source of increased EPA with DHA feeding is from ALA and not DHA. Although the liver is believed to be the major site of PUFA biosynthesis and retroconversion these results may be limited to liver EPA determinations only, and as an endpoint measure in a dynamic tissue may not fully represent values determined on a whole-body or specific tissue level. Based on our values, previous determinations of apparent retroconversion may be overestimated as a smaller proportion of the increase in liver EPA than previously reported may be attributed to DHA retroconversion. Moreover, the contribution of DHA to EPA may be further overestimated as additional ^13^C enrichment via preferential β-oxidation of ^12^C carbon and/or ^13^C-enriched acetyl-CoA from glycolysis may be present. In conclusion, the rate of retroconversion of DHA to EPA following dietary DHA feeding appears to be a minor contributor, with the majority of EPA derived from the elongation and desaturation of ALA.

## Abbreviations

ALA: α-linolenic acid
ARA: Arachidonic acid
CSIA: Compound specific isotope analysis
DHA: Docosahexaenoic acid
DPAn-3: Omega-3 docosapentaenoic acid
Elovl: Elongation of very long-chain
EPA: Eicosapentaenoic acid
FAME: Fatty acid methyl ester
GC-FID: Gas chromatography-flame ionization detection
IRMS: Isotope ratio mass-spectrometry
LNA: Linoleic acid
PUFA: Polyunsaturated fatty acid
TLE: Total lipid extract
